# Percutaneous fixation of traumatic pubic symphysis diastasis using a TightRope and external fixator versus using a cannulated screw

**DOI:** 10.1186/s13018-016-0397-7

**Published:** 2016-05-27

**Authors:** Yongzeng Feng, Jianjun Hong, Xiaoshan Guo, Chuangxin Lin, Wei Ling, Lifeng Zhang, Gang Wang

**Affiliations:** Department of Orthopaedic Trauma, Nanfang Hospital, Southern Medical University, No.1838, Guangzhou Road, Guangzhou, Guangdong Province 510000 China; Department of Orthopaedics Surgery, The Second Affiliated Hospital of Wenzhou Medical University, No.109, Xue Yuan West Road, Wenzhou, Zhejiang Province 325027 China; Department of Orthopaedics Surgery, The Third Affiliated Hospital of Southern Medical University, No.183, Zhongshan Road, Guangzhou, Guangdong Province 510000 China

**Keywords:** Symphysis pubis diastasis, Percutaneous reduction, TightRope fixation, Cannulated screw, External fixation, Comparative study

## Abstract

**Background:**

The aim of the study was to introduce a new percutaneous technique for the treatment of traumatic pubic symphysis diastasis using a TightRope and external fixator. A comparison between this technique and percutaneous fixation using a cannulated screw was performed.

**Methods:**

From January 2009 to December 2013, 26 patients with type II traumatic pubic symphysis diastasis were treated at two level 1 regional trauma centers. Among them, 10 patients were treated with a percutaneous TightRope and external fixator and 16 patients were treated with percutaneous cannulated screw fixation. Functional outcomes were evaluated using the Majeed scoring system. Patient satisfaction was evaluated using the modified visual analog scale. Radiological results were assessed based on the width of pubic symphysis preoperatively, immediately postoperatively, and at the final follow-up. Postoperative complications were also recorded.

**Results:**

There were no significant differences between the groups in Majeed scores and patient satisfaction (*p* > 0.05). There were no significant differences in the width of pubic symphysis preoperatively, immediately postoperatively, and at the final follow-up (*p* > 0.05). No significant differences were found regarding infection, fixation failure, or the need for revision surgery (*p* > 0.05).

**Conclusions:**

The new percutaneous technique using a TightRope and external fixator is a successful alternative for the treatment of type II traumatic pubic symphysis diastasis, which results in similar outcomes comparing to percutaneous cannulated screw fixation.

## Background

Pubic symphysis diastasis (PSD) is typically associated with a high-energy mechanism of injury [1]. Although many treatments have been reported on restoration of the anatomy of pubic symphysis, the appropriate strategy remains controversial [2, 3].

PSD is often a result of anterior-posterior compression (APC) injury based on the Young and Burgess classification system. The injuries are classified into three types, i.e., APC-I (slight widening of pubic symphysis or anterior sacroiliac joint, or both); APC-II (widened anterior sacroiliac joint >2.5 cm, disrupted anterior sacroiliac, sacrotuberous, and sacrospinous ligaments, intact posterior sacroiliac ligaments); and APC-III (complete sacroiliac joint disruption with lateral displacement, disrupted anterior sacroiliac, sacrotuberous, and disrupted posterior sacroiliac ligaments) [4–6].

APC-II injuries are rotationally unstable. Open reduction and internal fixation using plate and screw system has been widely accepted as the standard treatment [7, 8]. However, the technique needs extensive exposure of the pubic symphysis, resulting in more complications, such as blood loss, neural and vascular injuries, wound problems, and heterotopic bone formation [9–11]. In addition, the physiological movement across the pubic symphysis often leads to fixation failure [12].

**Fig. 1:**
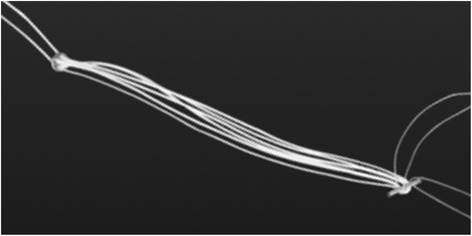
TightRope (Arthrex) device consists of a circular-shaped button and an oblong-shaped button. The buttons are connected with FiberWire threads

In order to reduce the complications, several other techniques have been reported, including external pelvic fixation, percutaneous cannulated screw fixation, minimally invasive plate osteosynthesis, and Endobutton technique for dynamic fixation [2–6]. In our hospital, percutaneous cannulated screw fixation is the common surgical procedure in the past 10 years. Since 2009, we have performed a new percutaneous dynamic fixation technique for PSD using a TightRope (Arthrex, Naples, FL, USA) device and external fixator (Fig. 1).

The purpose of the study was to introduce the new percutaneous technique for the treatment of APC-II traumatic PSD using a TightRope and external fixator. In order to better understand the procedure, we performed a comparison between this technique and percutaneous fixation using a cannulated screw [13].

## Methods

The study was approved by the Ethical Review Boards of Nanfang Hospital, Southern Medical University, and the Second Affiliated Hospital of Wenzhou Medical University. Informed consent for participation was obtained from all the patients.

From January 2009 to December 2013, a total of 35 consecutive patients with APC-II traumatic PSD were treated at two level 1 regional trauma centers. Inclusion criteria were adult patients who had a closed APC-II injury. The exclusion criteria were as follows: an APC-I PSD was excluded because the injury is often treated conservatively; an APC-III injury was excluded because combined posterior fixation is needed; patients who had lateral compression, vertical shear or combined mechanism fractures, open fractures, combined acetabular fractures, or pubic rami fractures were excluded; patients who had immature skeletons or medical contraindications, such as combined neurovascular injuries or diabetes, were excluded. Thus, 26 patients were included in the study and randomly allocated to group A (percutaneous fixation using a TightRope and external fixator) and group B (percutaneous fixation using a cannulated screw). The operator and tester were blinded.

**Fig. 2:**
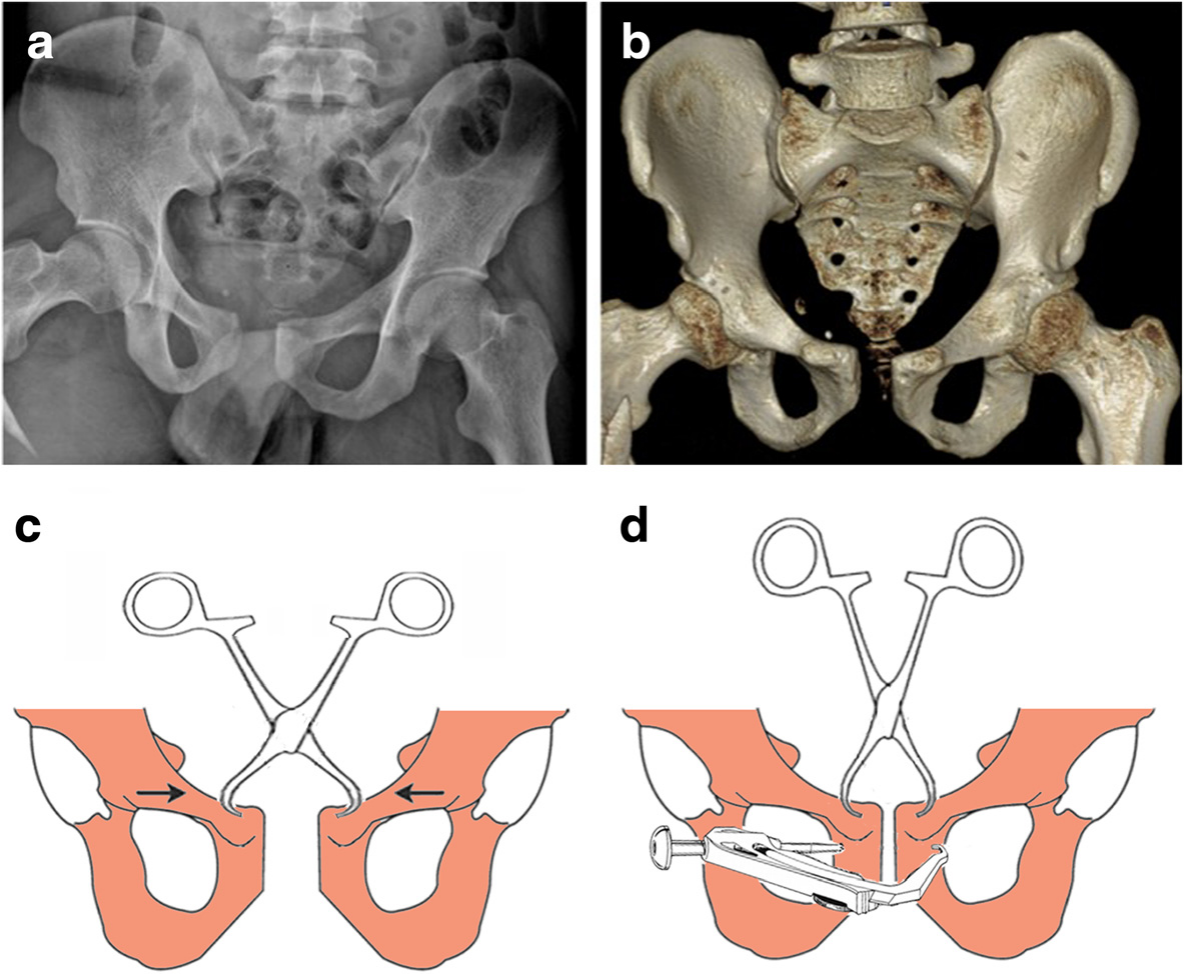
A male patient suffered pubic symphysis diastasis in a road traffic accident. **a** Preoperative anteroposterior radiograph. **b** Three-dimensional CT. **c** Reduction is maintained using pointed reduction clamps. **d** The insertion and exertion of the guide wire are located using a drill guide

Preoperatively, radiographs and CT scans were obtained to assess patterns of the injuries (Fig. 2a, b). Three patients underwent early pelvic angiography and embolization to stabilize their blood pressure.

### Surgical techniques

#### Group A

**Fig. 3:**
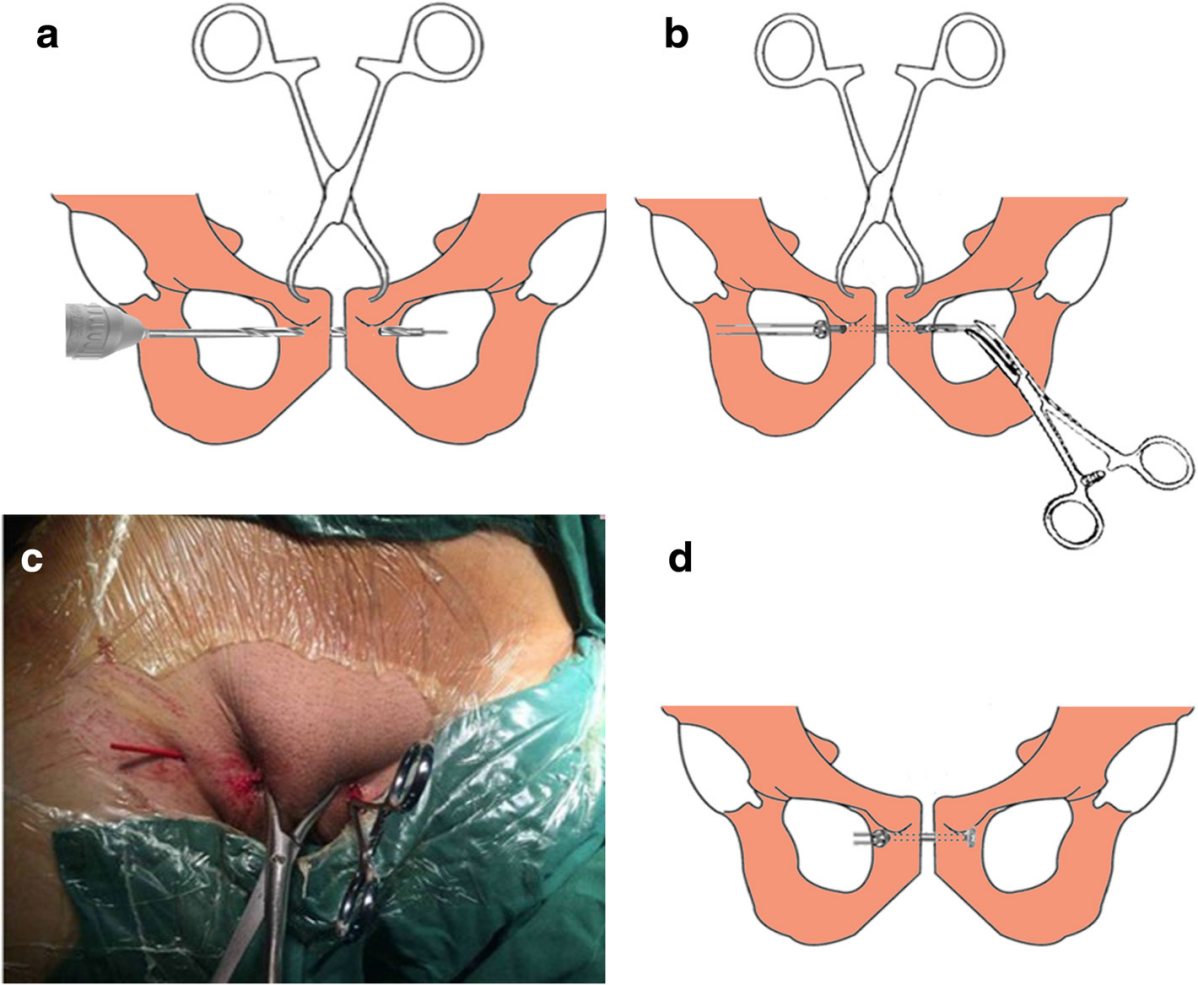
**a** A tunnel is created using a cannulated drill bit introduced over the guide wire. **b** The oblong-shaped button is passed through the tunnel. **c** A hollow tube was used across the traction thread. **d** The free ends of the FiberWire suture are tightened

The patient was placed in the supine position, and skin preparation was done. Two Schanz pins were inserted into either side of the iliac crest. Under C-arm fluoroscopic monitoring, the symphysis was reduced by manipulating the pins, and reduction was maintained percutaneously using pointed reduction clamps applying on the pubic tubercles (Fig. 2c). Utilizing a matching drill guide, the insertion and exertion points were located at the juncture between the pubic tubercle and obturator on either side (Fig. 2d). A 1.5-mm guide wire was drilled transversally across the symphysis. Correct wire positioning was confirmed under anteroposterior, outlet, and inlet views. Care was taken not to injure the spermatic cord in male patients and the round ligament of uterus in female patients. A bony tunnel was created using a 4.0-mm cannulated drill bit introduced over the guide wire (Fig. 3a). Then, a 3.0-mm hollow tube was inserted through the drilled tunnel, in which a traction thread was introduced in advance. The oblong button of TightRope was passed through the drilled tunnel to the opposite side by pulling the traction thread using a hemostat (Fig. 3b). The tube was removed, and both buttons were seated flush in the correct position (Fig. 3c). The FiberWire suture at the skin insertion was tightened manually (Fig. 3d). Finally, an external fixator was fixed on the Schanz pins to reinforce pelvic stability.

#### Group B

As described by Chen et al. [3], the symphysis was stabilized percutaneously using a 6.5-mm cannulated lag screw. No additional external fixation was used.

### Postoperative managements

**Fig. 4:**
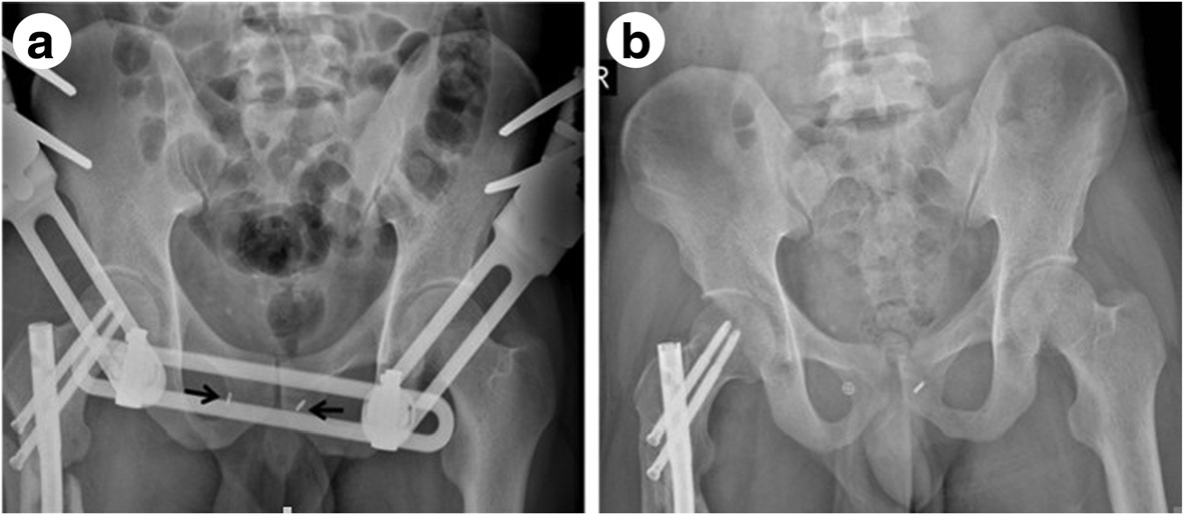
Postoperative radiographs. **a** Immediately after surgery. **b** One year after surgery

Patients were encouraged to perform non-weight-bearing exercises when pain could be tolerated. The external fixator in group A was removed 6 weeks after surgery. Partial weight bearing was allowed at that time and progressed gradually according to the condition of each patient’s associated injuries. All patients were followed up at 6 weeks, 3 months, 6 months, and 12 months postoperatively and annually thereafter (Fig. 4).

### Outcome evaluation

Functional outcomes were evaluated using the scoring system described by Majeed [14], including pain, sitting, sexual intercourse, walking, and work. Patient satisfaction was evaluated using the modified visual analog scale based on a range from 0 to 10, with 0 indicating maximum dissatisfaction and 10 indicating full satisfaction. Postoperative complications were also recorded.

The width of the pubic symphysis was measured preoperatively, immediately after surgery, and at the final follow-up using the picture archiving and communication system (Infinitt, Seoul, South Korea) [15]. Fixation failure was defined as loosening or breakage of Endobutton device or screw.

### Statistical analysis

Statistical analysis was performed with SPSS 17.0 software for Windows. Continuous data with a normal distribution were expressed as the mean ± standard deviation. Continuous variables with non-normal distributions were analyzed with the Mann-Whitney *U* test. Non-paired *t* test was used for comparison of continuous data that appeared to be approximately normally distributed. Categorical data were statistically analyzed using Fisher’s exact test (*n* < 40 or *t* < 1). A *p* value of <0.05 was considered statistically significant.

## Results

All 26 patients were followed for an average of 15 months (range, 12–20 months). We found no significant differences in patient age, sex, injury mechanism, and time from injury to operation between the two groups (*p* > 0.05). The operative time of group A was 48.5 ± 9.4 min, which was significantly longer than that of group B (27.3 ± 5.1 min) (*p* < 0.001; Table 1). One patient in group A and two patients in group B had urethral injuries. As a result, one patient was treated conservatively and two other patients underwent surgery. Two patients in group A and three patients in group B had cerebral contusions or thoracic injuries, or both, and all of them were treated conservatively. Four patients in group A had combined femoral (*n* = 2), tibiofibular (*n* = 1), and lumbar (*n* = 1) fractures. Five patients in group B had combined femoral (*n* = 3), forearm (*n* = 1), and lumbar (*n* = 1) fractures. Those fractures were simultaneously treated with open or closed reduction and internal fixation, and bone healing was achieved in all cases.

**Table 1:** Patient characteristics and surgical details of the two groups (*x* ± *s*)

	Group A	Group B	*p* value
	(*n* = 10)	(*n* = 16)	
Age (year)	32.5 ± 6.2	33.2 ± 5.8	0.781
Sex (m/f)	7/3	11/5	1.000
Injury mechanism (*n*)			1.000
Traffic accident	8	13	
Crushing	2	3	
Time to operation (day)	4.8 ± 2.9	4.1 ± 3.0	0.580
Operative time (min)	48.5 ± 9.4	27.3 ± 5.1	0.000
Width of pubic symphysis (mm)			
Preoperation	40.0 ± 12.3	41.4 ± 10.4	0.813
Immediately postoperation	4.1 ± 0.6*	4.5 ± 0.7**	0.118
Final follow-up	4.3 ± 0.5***	4.7 ± 0.7****	0.113
Complications (*n*)			
Infection	1	0	0.385
Fixation failure	1	3	1.000
Revision surgery	0	1	1.000
Satisfaction (MVAS)	8.1 ± 1.2	8.4 ± 1.0	0.538
Majeed score (*n*)			0.897
Excellent (>85)	7	11	
Good (70–84)	3	4	
Fair (55–69)	0	1	
Poor (<55)	0	0	

*MVAS* modified visual analog scale

*p = 0.000; **p = 0.000; ***p = 0.419; ****p = 0.445

Preoperatively, the width of pubic symphysis was 40.0 ± 12.3 mm in group A and 41.4 ± 10.4 mm in group B. Immediately after surgery, the width decreased to 4.1 ± 0.6 and 4.5 ± 0.7 mm in groups A and B, respectively (*p* < 0.001). At the final follow-up, the width was 4.3 ± 0.5 and 4.7 ± 0.7 mm, respectively. In both groups, the width of pubic symphysis was significantly decreased immediately after surgery (Pa = 0.000, Pb = 0.000), but no significant difference was found between the data measured immediately after surgery and at the final follow-up (Pc = 0.419, Pd = 0.445) (Table 1). We found no significant difference between the groups in preoperative or immediately postoperative data, nor data measured at the final follow-up.

One patient in group A developed a superficial infection at the skin insertion. One patient in group A and three patients in group B had a fixation failure. One patient in group B had a revision surgery. No significant difference was found regarding infection, implant failure, or revision surgery between the two groups. We found no significant difference in Majeed scores or patient satisfaction between the groups (Table 1). Detailed results and scores regarding pain, sitting, sexual intercourse, walking, and work are shown in Table 2.

**Table 2:** Detailed scores according to the Majeed scoring system at the final follow-up

Group/no.	Pain	Sitting	Sexual intercourse	Walking	Work	Total	Grade
A							
1	30	8	4	34	16	92	Excellent
2	30	10	4	36	16	96	Excellent
3	25	8	3	34	16	86	Excellent
4	25	12	3	28	16	84	Good
5	30	10	4	34	16	96	Excellent
6	20	6	4	28	16	74	Good
7	25	8	4	32	20	89	Excellent
8	25	6	3	32	16	82	Good
9	30	10	4	34	20	98	Excellent
10	25	10	4	34	20	93	Excellent
B							
1	25	8	4	32	20	89	Excellent
2	25	10	4	34	20	93	Excellent
3	30	10	4	36	16	96	Excellent
4	20	8	4	34	16	82	Good
5	25	10	4	32	20	91	Excellent
6	20	6	4	28	16	74	Good
7	25	8	3	32	12	80	Good
8	25	10	4	36	20	95	Excellent
9	25	8	4	30	16	83	Good
10	30	8	4	36	20	98	Excellent
11	20	6	3	28	12	69	Fair
12	25	10	4	30	20	89	Excellent
13	30	10	4	34	16	94	Excellent
14	30	10	4	36	12	92	Excellent
15	25	10	4	36	20	95	Excellent
16	25	8	4	36	16	89	Excellent

## Discussion

PSD may occur alone or be combined with other pelvic fractures. According to APC in the Young-Burgess classification of pelvic disruption, a diastasis of more than 2.5 cm is classified as an APC-II injury pattern. It is a common “open book” injury with rotational instability. It is routinely treated surgically [16]. We found that our percutaneous technique using a TightRope and external fixator is a safe, minimally invasive, dynamic fixation for the treatment of APC-II traumatic PSD.

In adults, the pubic symphysis has small-magnitude, multidirectional movements under physiological conditions [17, 18]. The traditional static immobilization using a plate and screw system carries a high rate of fixation failure [19]. The reported percentage is above 30 %. Implant loosening and breakage may result in the recurrence of PSD and pain [12]. Chen et al. reported percutaneous cannulated screw fixation. Comparing to the plate fixation, the cannulated screw fixation can also well maintain the reduction. The minimally invasive technique has advantages of a lower risk of iatrogenic injuries, more rapid recovery, and better appearance. However, the screw fixation is also a static immobilization, which carries the similar fixation failure [3]. Chen et al. also evaluated 21 patients with PSD undergoing Endobutton (Smith and Nephew, Memphis, TN, USA) fixation. The results indicate that Endobutton fixation might be an alternative treatment for APC-II injuries. However, they used it through open reduction. The length of the loop in Endobutton is specified, and it cannot be tightened freely as needed [6].

In order to decrease the complications, we introduced the dynamic percutaneous fixation technique using a TightRope device. The device was originally designed for stabilization of tibiofibular syndesmosis [20]. It consists of two metal buttons, circular and oblong buttons, joined by a continuous loop of FiberWire suture. It can be tightened manually and does not need to be tied in a knot. It has also been indicated in acromioclavicular dislocation, distal clavicle fractures, and anterior cruciate ligament reconstruction [21–23]. TightRope provides semi-rigid fixation that allows minor physiological motion, which decreases the stress on the implant. The additional external fixation adds more stability in the early postoperative stage.

The best indication of percutaneous TightRope technique might be APC-II injury or type B1 or B2 (according to the Tile classification). Increased clinical experience and biomechanical studies about TightRope may make it easier to use in more complicated cases, such as APC-III injuries. Contraindications are open pelvic fractures, combined acetabular fractures, pubic rami fractures, and medical contraindications.

Advantages of the technique are a minimally invasive procedure, less blood loss, minimal complications, and rapid recovery. The dynamic fixation decreases stress at the symphysis pubis during its ligament healing and micro-movement, which decreases the risk of implant failure. Implant removal is not necessary. The disadvantages are a complex procedure, and the operation time is relatively longer.

The present study has some limitations. The small number of patients weakens the statistical power of the final results. Further investigation in a larger sample size and longer follow-up time is needed to obtain more overall clinical data. In addition, the differences in surgeons’ performance and patients’ associated injuries may have decreased the generalization power of this study. Furthermore, biomechanical research on the technique is necessary. Despite all these limitations, the present study contributes significantly to the current treatment of pubic symphysis diastasis.

## Conclusions

The dynamic percutaneous technique using a TightRope and external fixator is a minimally invasive procedure with minimal morbidity. Both the TightRope device and the cannulated screw are effective in the treatment of APC-II traumatic PSD.
